# Time to Regenerate: Ecosystems and Health Promotion

**DOI:** 10.1371/journal.pmed.0030394

**Published:** 2006-10-24

**Authors:** Colin D Butler, Sharon Friel

## Abstract

Butler and Friel call upon the health promotion community to strongly reengage with environmental and ecological issues.

The concept of health promotion gradually emerged from the discipline of public health during the 1970s. In the mid-19th century, founders of the growing discipline of public health had stressed the importance of social, political, and environmental factors as key determinants of disease and health. However, as microbiology and epidemiology developed, and as the most egregious examples of environmental and social abuse faded from view in developed countries, emphasis on these factors waned within mainstream public health, which remained best established in industrialised countries.

As globalisation gathered momentum, and as decolonisation proceeded, interest in social determinants of health re-surfaced [1], culminating in the primary health care (PHC) movement [2–4] and the seminal Health for All declaration. The declaration, made at the 1978 International Conference on Primary Health Care convened in Alma Ata, Kazakhstan, included the statement that “an acceptable level of health for all the people of the world by the year 2000 can be attained” [5].

The PHC movement called for the balancing of purely medical aspects of health care with greater emphasis upon the social, economic, and political determinants of health, particularly for those members of the global population whose income was low. Eight years later, in November 1986, the Ottawa Charter was signed, and this remains the best-known declaration of the principles of health promotion [6]. This charter asserted that environmental and ecological factors are of fundamental importance for health.

Since 1986, the evidence linking health to ecological and environmental factors (such as climate change, biodiversity loss, and the mental health benefits of exposure to nature) has strengthened considerably, stimulating a new discipline, sometimes called “ecohealth” (see Sidebar). However, we believe that paradoxically, recognition of the importance of environmental and ecological factors has simultaneously declined among proponents of health promotion.

**Sidebar: Ecohealth**

Ecohealth extends traditional environmental health by studying the relationship between health and explicitly ecological factors such as biodiversity and ecosystem “services” [27]. There are four kinds of services: “provisioning” (e.g., food), “regulating” (e.g., climate), “cultural” (e.g., sacred groves), and “supporting” (e.g., the maintenance of soil fertility by worms). More subtly, ecohealth borrows insights developed by human ecology to understand and predict health through consideration of the relationships between human populations and between human and non-human species. At the largest scale, ecohealth differs conceptually from traditional environmental health in considering humans as a part of the global biosphere—the systemic, interacting forces which regulate life and its inorganic substrate [28]. Falling within this scope are topics such as health and the global atmosphere, including climate change, stratospheric ozone depletion, and the movement of transcontinental air pollution and dust clouds. Even more broadly, ecohealth grapples with the sustainability of civilisation, and therefore of human health [11].

This essay traces and analyses the growing separation between health promotion and ecohealth. We call upon health promotion advocates to strongly re-engage with environmental and ecological issues, and to form active alliances with advocates of ecohealth. We believe this will strengthen health promotion, benefit ecohealth, and help to promote and protect sustainable global health.

## Health Promotion

Health promotion seeks to understand and address the complex constellation of social, environmental, and political factors that underpin health. These factors, often described simply as the “social determinants of health” [1], are “upstream” of more obvious proximal causes of health outcomes. This distance upstream can be temporal or conceptual.

The Alma Ata conference identified two of eight essential components of PHC as environmental: adequate nutrition and safe water, and basic sanitation. The Ottawa Charter, regarded as a milestone in the development of health promotion, paid even more attention to environmental issues than did Alma Ata. The charter emphasised the role of a “stable ecosystem” and sustainable resources as fundamental conditions and resources for health. The Charter declared: “The fundamental conditions and resources for health are peace, shelter, education, food, income, a stable eco-system, sustainable resources, social justice and equity” [6].

Of these nine fundamental elements, three (food, a stable ecosystem, and sustainable resources) are directly related to environmental factors, while three others (peace, social justice, and equity) often depend on the distribution of environmental and ecological resources. The Charter further stated: “The conservation of natural resources throughout the world should be emphasized as a global responsibility... Systematic assessment of the health impact of a rapidly changing environment—particularly in areas of…energy production and urbanization—is essential…The protection of the natural and built environments and the conservation of natural resources must be addressed in any health promotion strategy” [6].

## Health Promotion, Ecohealth, and Global Environmental Change

An understanding of the effect of the physical environment on health is ancient, and environmental health has been central to public health since its foundation in the mid-19th century. However, the concept of “ecohealth” is far more recent (see Sidebar) [7].

The central concepts of ecohealth seem to have been well understood by the drafters of the founding documents of both health promotion and PHC [8]. Foreshadowing the “Earth Summit” held in Rio de Janeiro in 1992, Maurice King, a pioneer of the PHC movement, called upon the World Health Organization (WHO) in 1990 to incorporate the word “sustainable” into its definition of health [9]. Concerns about global environmental change persisted within the health promotion movement in its early years (see Figure 1). The Third International Conference on Health Promotion, convened by WHO in Sundsvall, Sweden in 1991, stressed the importance of environmental health, stating: “The issues of health, environment and human development cannot be separated. Development must imply improvement in the quality of life and health while preserving the sustainability of the environment” [10].

**Figure 1:**
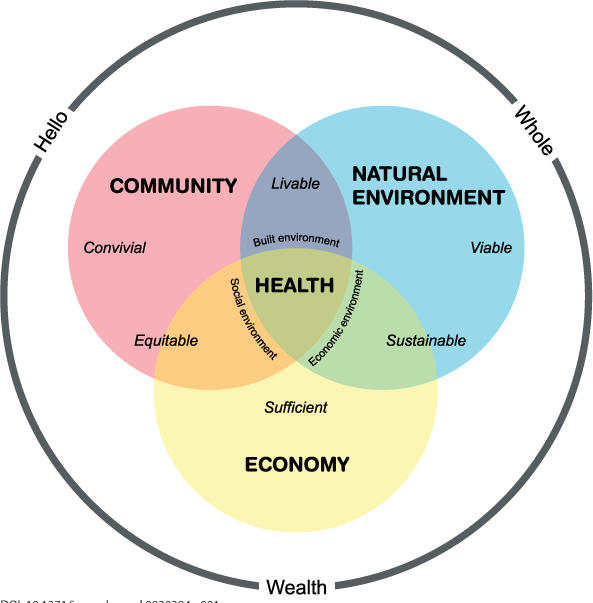
Labonté's 1993 Description of a Holosphere of Healthy Communities, with Health at the Centre (Derived from [8]). The interacting environmental spheres include a viable and sustainable natural environment and a sustainable economy.

Since the Earth Summit, the evidence for adverse environmental change at the global scale has strengthened alarmingly [11–14]. Humanity has overloaded the Earth's capacity to absorb waste and damaged many ecosystem “provisioning” services, such as pristine water and fisheries [12]. The reality and potential severity of ongoing climate change is now widely accepted. Erosion and other forms of soil damage, increasing oil scarcity, and the still expanding global population combine to place food security at risk, particularly in Africa [15,16]. Ample food to eradicate human hunger has been grown for a long time, but worsening maldistribution of food means the absolute number of hungry people is again rising [17].

While adverse global environmental change has not yet translated to an unequivocal decline in human health, fears that this may happen are increasingly credible [13]. Yet, with a few exceptions [18,19], ecohealth seems now to be peripheral to the health promotion agenda. This is reflected in the Bangkok Charter signed at the Sixth Global Conference on Health Promotion in 2005 [20].

The gap between health promotion and ecohealth is especially striking because health promotion claims to focus on causally important factors, even when their link to health is lagged, complex, or theoretical [21]. Many aspects of global environmental change that fit this category are worsening, but are currently largely ignored by health promotion.

## Explaining the Gap between Health Promotion and Ecohealth

Since the Ottawa Charter was signed, many of the social and economic underpinnings of population health (such as the gap between rich and poor) have also deteriorated, apart from those that are purely environmental. Health promotion has continued to champion the issue of equity in the face of greater international and domestic inequality [11,22] but has largely abandoned its stress on the linkages between adverse global environmental change and human health.

This separation is intriguing. All social movements and scientific disciplines are subject to powerful institutional and natural forces that shape their social, economic, political, and environmental milieu, and it is unremarkable that a reformist movement such as health promotion is not always successful in meeting its goals. One possible factor which may explain the disturbing separation between health promotion and ecohealth is the close relationship between WHO and the health promotion movement [23]. This closeness may have seemed a boon for the new discipline, promising a mutually interactive and progressive relationship, in which the resources of WHO would shelter and support health promotion, and in which health promoters could tickle the conscience of WHO.

However, like the health promotion movement, WHO is also subject to larger forces. Since Alma Ata, the rhetoric, aspiration, influence, and—arguably—the achievement of WHO has diminished, coincident with a decline in many public goods [24]. Neither WHO nor its constituent national governments have given the issue of adverse global environmental change the attention it deserves, though this might be starting to change. The 1992 Earth Summit received enormous fanfare yet, we argue, achieved little. Although the participants called for a fundamental transformation of the global socio-political landscape, with hindsight it is obvious that rich populations were unwilling to make the necessary sacrifices to protect future generations, when “business as usual” could ensure a short-term continuation of prosperity. Such ideas could, of course, never be expressed as frankly as we are describing them here, and they still rarely are. However, we suggest that this “business as usual” approach affected WHO and its constituent governments. Sustainability was to be honoured by promises rather than action. Gradually, we believe, these norms came to permeate the health promotion movement as well as the fields of public health and epidemiology.

We are not suggesting that the failure of health promotion to address the challenge of ecohealth, and especially its dimension of global environmental change, lies entirely or even mainly with WHO. Rather we emphasise the view that powerful forces embedded and reflected in the norms, laws, and customs of humanity have overwhelmed the capacity of health promotion proponents to recognise, analyse, and address adverse global environmental change.

Of course, these statements are generalisations. There are some welcome signs of change, both within national governments and WHO [12] and within the health promotion movement. However, this awakening is insufficient given the immensity of the problems. It is of vital importance that health promotion return to its roots and join the forefront of this movement.

## Revitalising Health Promotion by Integrating it with Ecohealth

Although the field of ecohealth has only recently emerged, it has many roots in public health and health promotion as originally conceived. There are obvious potential synergies between ecohealth and health promotion. Indeed, arguably, ecohealth has arisen to fill the vacuum created by health promotion's abandonment of ecology. Curiously, though with some exceptions, ecohealth seems almost as light on its analysis of power and inequality as health promotion now is with regard to ecological issues. This conceptual oversight is partly explained by the narrow focus of most discipline-based researchers, who are rarely trained or encouraged to venture into other disciplinary territories. We also suggest that a central problem in the rhetoric of sustainability is its reticence in calling for fundamental changes to existing power structures.

The strategy of most sustainability activists, especially those based in wealthy countries, has been timid. The 1992 Earth Summit called for a fundamentally fairer world order, but few Western participants took this seriously. It was at that meeting that US President George Bush famously stated that the lifestyle of the average American was not open to negotiation. Environmentalists often call for ecological and climatic protection for its own sake, but rarely discuss the really difficult personal, social, and economic actions necessary to achieve this. Finally, very few environmentalists are genuinely engaged with the struggle to reduce global poverty. In short, just as health promotion could learn from ecohealth, so too could ecohealth learn from health promotion.

We propose three concrete steps to revitalise health promotion. The success of these suggestions depends on a more receptive international milieu. However, as environmental harm intensifies, and as the price of oil climbs ever higher, it is plausible that this milieu will become much more favourable to the quest for sustainability. The discipline and goals of ecohealth will benefit from an interaction with health promotion, especially if ecohealth can use some of the insights and strategies developed and used by health promotion to counter the marketing of products that are harmful to health, such as tobacco.

First, we suggest that a reinvigoration of the Healthy Cities movement, with an equity focus, is keenly needed. This movement, sometimes traced to Toronto in 1984, is an attempt to foster friendlier and less environmentally damaging urban settings, which, consequently, will be healthier and more sustainable. There are potential synergies in addressing the challenges of climate change, oil depletion, unsustainable agriculture, the “obesogenic” environment, and improving communication and energy technology. (The obesogenic environment refers to settings that encourage excess caloric intake through the use of fossil fuel–powered devices rather than the expenditure of human muscular energy, coupled with excess availability of energy-dense, nutrient-empty foods). Such synergies could stimulate a large market not only for more sustainable urban areas, but also for healthier villages and communities.

Second, the movements of ecohealth and health promotion combined may have sufficient clout at a global level to nurture a “coalition of the giving” between the G-8 and large developing countries in order to accelerate the new technologies desperately needed to slow climate change and ease the growing energy crisis. Increased funding, permitted by this more favourable milieu, would allow a large increase in the employment of practitioners with training and experience in these issues, trained by improved curricula, and supported at the local level by infrastructure which encourages sustainability. Similar to health impact assessments, “sustainability impact statements” could be mandatory for all new developments.

Third, reducing global poverty and inequality will dramatically slow population growth, and thus greatly enhance population health [15,25]. Ecohealth and health promotion combined could advance awareness and action concerning this issue.

## Conclusion

When Katherine Mansfield wrote, “By health I mean the power to live a full, adult, living, breathing life in close contact with what I love” [26], she inadvertently captured the political, environmental, social, and personal context in which health promotion finds itself in the 21st century.

The knowledge and methods developed by health promoters to advance social change to improve health can and should be used to promote the social changes needed to promote ecohealth. Such promotion will have symbiotic benefits for the health of human populations and the state of the physical environment. The challenge is to ensure government commitment to health-promoting policies, whatever they are called, and to advance partnerships between the new and old health players. Health promotion cannot abandon its pursuit of social justice. Without sustainability, neither health nor social justice can be attained.

## Abbreviations

PHC: primary health care
WHO: World Health Organization
